# Tuberculosis-associated anemia is linked to a distinct inflammatory profile that persists after initiation of antitubercular therapy

**DOI:** 10.1038/s41598-018-37860-5

**Published:** 2019-02-04

**Authors:** Leonardo Gil-Santana, Luís A. B. Cruz, María B. Arriaga, Pryscila F. C. Miranda, Kiyoshi F. Fukutani, Paulo S. Silveira-Mattos, Elisangela C. Silva, Marina G. Oliveira, Eliene D. D. Mesquita, Anneloek Rauwerdink, Frank Cobelens, Martha M. Oliveira, Afranio Kritski, Bruno B. Andrade

**Affiliations:** 1 Instituto Gonçalo Moniz, Fundação Oswaldo Cruz, Salvador, Brazil; 2 Multinational Organization Network Sponsoring Translational and Epidemiological Research (MONSTER) Initiative, Fundação José Silveira, Salvador, Brazil; 3 Curso de Medicina, Faculdade de Tecnologia e Ciências, Salvador, Brazil; 4 Tuberculosis Academic Program, Medical School, Federal University of Rio de Janeiro, Rio de Janeiro, Brazil; 5 Universidade Salvador (UNIFACS), Laureate Universities, Salvador, Brazil; 6 Recognize the Biology Laboratory, Center of Bioscience and Biotechnology, State University of North Fluminense Darcy Ribeiro, Rio de Janeiro, Brazil; 7 Ary Parreira Institute, State Secretary of Health of Rio de Janeiro, Rio de Janeiro, Brazil; 8 Amsterdam Institute for Global Health and Development, Amsterdam University Academic Medical Centre, Amsterdam, The Netherlands; 9 Centro de Desenvolvimento Tecnológico em Saúde (CDTS), Fundação Oswaldo Cruz, Rio de Janeiro, Brazil; 10 Escola Bahiana de Medicina e Saúde Pública (EBMSP), Salvador, Brazil

## Abstract

Pulmonary tuberculosis (PTB) is associated with chronic inflammation and anemia. How anemia impacts systemic inflammation in PTB patients undergoing antitubercular therapy (ATT) is not fully understood. In the present study, data on several blood biochemical parameters were retrospectively analyzed from 118 PTB patients during the first 60 days of ATT. Multidimensional statistical analyses were employed to perform detailed inflammatory profiling of patients stratified by anemia status prior to treatment. Anemia was defined as hemoglobin levels <12.5 g/dL for female and <13.5 g/dL for male individuals. The findings revealed that most of anemia cases were likely caused by chronic inflammation. A distinct biosignature related to anemia was detected, defined by increased values of uric acid, C-reactive protein, and erythrocyte sedimentation rate. Importantly, anemic patients sustained increased levels of several biochemical markers at day 60 of therapy. Preliminary analysis failed to demonstrate association between persistent inflammation during ATT with frequency of positive sputum cultures at day 60. Thus, TB patients with anemia exhibit a distinct inflammatory profile, which is only partially reverted at day 60 of ATT.

## Introduction

Tuberculosis (TB) remains the major cause of death from infection by a single pathogen^1^. *Mycobacterium tuberculosis* infection drives a chronic pulmonary disease characterized by persistent granulomatous inflammation with substantial lung tissue damage^2^. The chronic inflammation observed in pulmonary TB patients is reflected by increased circulating levels of acute phase proteins, such as C-reactive protein (CRP) as well as of inflammatory cytokines^3,4^. In fact, patients with more severe clinical forms of TB disease have been shown to exhibit a distinct inflammatory profile associated with balance between different cytokines and lipid mediators^5^. Understanding the mechanisms driving increased susceptibility to persistent pathological inflammation in TB may help development of new treatment strategies to optimize patient care.

Many patients with active pulmonary TB exhibit decreased levels of hemoglobin, which can directly impact TB-associated morbidity. Anemia can be defined as hemoglobin (Hb) levels below 12.5 g/dL for women and 13.5 g/dL for men^6^. Anemia can have many causes, including iron deficiency and chronic inflammation. These two distinct mechanisms of anemia present different laboratorial definitions. Anemia caused by iron deficiency is associated with ferritin levels <30 ng/mL whereas that caused by chronic disease is linked to ferritin levels >100 ng/mL^7^. In addition, serum concentrations of ferritin are reported to increase in inflammatory conditions such as autoimmune diseases, infections, malignancy and other diseases^8–10^. In both types of anemia, transferrin saturation values drop below 20%^11^. Anemia has been extensively studied in the context of TB and both iron deficiency-related and chronic disease-associated anemias have been reported^12–15^. The specific interplay between systemic inflammation, mycobacterial loads in sputum and anemia status in cohort studies is unknown.

In the present study, we perform a detailed profiling of systemic inflammation in a cohort of active pulmonary TB patients presenting with or without anemia before and at different time points after antitubercular therapy (ATT) initiation. Our findings reveal a very distinct inflammatory profile in anemic patients. This pro-inflammatory state was only partially reduced after 60 days of ATT, indicating that TB-associated anemia is indeed related to persistent inflammation.

## Results

### Patients with TB exhibit a distinct profile of biochemical profiles in peripheral blood

**Figure 1:**
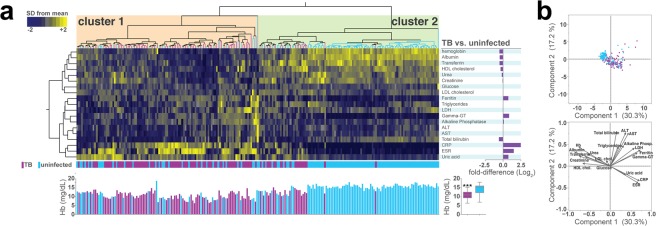
Pulmonary TB patients display a unique profile of circulating biochemical parameters of inflammation. (**a**) Upper panel: A hierarchical clustering analysis (Ward’s method) was employed to illustrate the overall profile of inflammatory parameters in pulmonary TB patients or healthy controls. Each column represents one patient. Fold differences (TB vs. healthy controls) were calculated and statistically significant differences are highlighted in purple. Lower panel: Histogram for individual values of hemoglobin (Hb). Distributions of Hb were compared between TB patients and healthy controls using the Mann-Whitney *U* test. ***p < 0.0001. (**b**) A principal component analysis (PCA) model was employed to test whether combination of the markers evaluated could cluster TB patients separately from controls. A vector analysis was utilized to illustrate the influence of each biochemical parameter in the distribution of the data of the PCA model. Markers in red indicate those with the highest fold-difference values between TB and healthy controls. Abbreviations: ALP, alkaline phosphatase; ALT, alanine transaminase; AST, aspartate aminotransferase; Gamma-GT, gamma-glutamyl transferase; HDL, high-density lipoprotein; LDH, lactate dehydrogenase; LDL, low-density lipoprotein; VLDL, very-low-density lipoprotein.

Initially, data from a total of 238 individuals were retrospectively examined. PTB patients were older and presented with lower BMI on average than the non-TB controls (Table 1). In addition, TB patients reported alcohol abuse, smoking and use of illicit drugs more frequently than controls (Table 1). HIV infection and previous TB treatment were both more frequently referred in TB patients compared to non-TB controls (Table 1). Numerous parameters were examined in peripheral blood samples from the study population. Hierarchical cluster analysis of z-score normalized data of these variables was performed to test if simultaneous assessment of the values from these markers could distinguish TB patients at pre-ATT from healthy controls (Fig. 1a). We found that pulmonary TB patients generally exhibited a distinct profile of gamma-glutamyl transferase (gamma-GT), and, expectedly, of CRP, erythrocyte sedimentation rate (ESR), uric acid and ferritin (Fig. 1a). On the other hand, healthy individuals exhibited predominantly increased values of hemoglobin, albumin, transferrin, as well as of HDL cholesterol, urea and total bilirubin levels (Fig. 1a). In addition, vector analysis integrated with a PCA model also demonstrated that heightened CRP, ESR and uric acid levels were more associated with active TB, whilst higher values of hemoglobin, albumin, transferrin, urea and creatinine hallmarked healthy controls (Fig. 1b).

**Table 1:** Characteristics of study population at the study enrollment.

Characteristic	TB patients	Controls	p-value
N	118	118*	
Male– no. (%)	91 (77.1)	112 (95.0)	<0.01
Median age– years (IQR)	40.5 (31–48)	25 (20–33)	<0.01
Median BMI– Kg/m² (IQR)	17.6 (16.3–20.0)	22.7 (20.5–25.7)	<0.01
Previous TB treatment– no. (%)**	42 (35.6)	7 (38.9)	>0.99
HIV– no. (%)**	12 (5.7)	5 (5.6)	0.06
**Lifestyle habits– no. (%)**			
Alcohol abuse**	71 (60.2)	18 (35.3)	<0.01
Smoking history**	84 (71.2)	36 (30.8)	<0.01
Illicit drugs use**	34 (28.8)	8 (7.1)	<0.01

Age and body mass index (BMI) were compared using the Mann Whitney *U* test. Frequencies of male and indicated lifestyle habits were compared using Fisher exact test. IQR, interquartile range. *118 from the 120 uninfected control subjects had all the epidemiological data available. **Variables presented different number of patients from which data was available for: data from 113 TB patients and 18 uninfected controls was available with regard to history of TB treatment, data from 99 TB patients and 14 uninfected controls was available under the variable HIV, data of 117 patients from both columns under the variable Smoking history and from 117 TB patients and 113 uninfected controls under the variable Illicit drugs use were available.

### Anemic TB patients present a distinct biochemical profile

Among the 118 TB patients 105 (88.9%) presented with Hb levels below the reference values for male or female individuals and were categorized as anemic (Table 2). Neither sex, age, BMI, alcohol abuse, smoking nor illicit drug use could distinguish anemic and non-anemic TB patients (Table 2).

**Table 2:** Characteristics of TB patients at the study enrollment.

Characteristic	Anemia	No anemia	p-value
N	105	13	
Male– no. (%)	82 (78.1)	9 (69.2)	0.49
Median age– years (IQR)	40 (30–51)	45 (23–50)	0.56
Median BMI– Kg/m² (IQR)	17.6 (15.5–20.7)	17.6 (13.3–24.4)	0.59
Previous TB treatment– no. (%)*	39 (37.1)	3 (23.1)	0.38
HIV– no. (%)*	19 (18.4)	0 (0)	0.12
**Lifestyle habits– no. (%)**			
Alcohol abuse*	66 (62.9)	5 (38.5)	0.13
Smoking History*	78 (74.3)	6 (46.2)	0.05
Illicit drugs use*	32 (30.5)	2 (15.4)	0.34
AFB Smear Grade– no. (%)			0.06
0	19 (18.10)	0 (0)	
1+	38 (36.19)	7 (53.85)	
2+	28 (26.67)	6 (46.15)	
3+	20 (19.04)	0 (0)	

Age and body mass index (BMI) were compared using the Mann Whitney *U* test. Data on AFB Smear Grade frequencies were compared using the Chi-Square test. Frequencies of male and indicated lifestyle habits were compared using Fisher exact test. IQR, interquartile range. *Variables presented different number of patients from which data was available for: data from 90 anemic patients and 23 non anemic patients was available with regard to history of TB treatment, data from 80 patients with anemia and 19 patients without anemia was available under the variable HIV, data from 24 patients without anemia was available for the variable Smoking history, and data of 92 patients was available from anemic patients under the variable Illicit drugs use.

**Figure 2:**
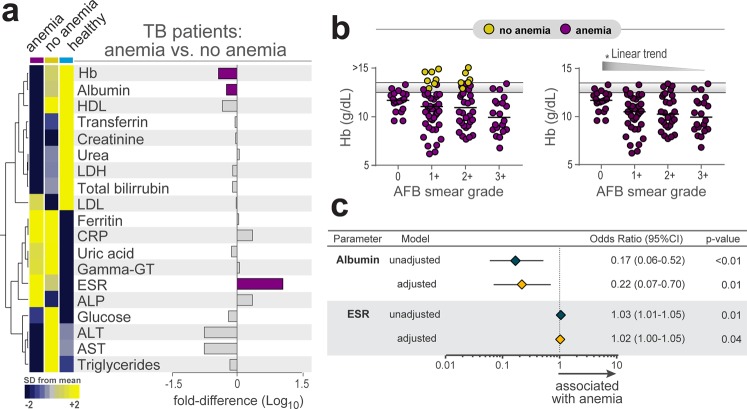
Biochemical differences between pulmonary TB patients with or without anemia before antitubercular treatment initiation. (**a**) Data on each parameter was Log10 transformed. Mean values for each indicated clinical group were z-score normalized and a Hierarchical cluster analysis was performed to illustrate the overall biochemical profiles. Fold differences (TB anemia vs. TB no anemia) were calculated and statistically significant differences are highlighted in purple. (**b**) Levels of hemoglobin in the in all TB patients (left panel) or only in anemic patients (right panel) stratified by AFB smear grade were compared using the Kruskal-Wallis test with non-parametric linear trend post hoc test. **p < 0.001. Yellow plots represent non-anemic TB patients, while purple plots represent anemic TB patients. Horizontal lines represent the two cut-offs for anemia for men and women. (**c**) Logistic linear regression model adjusted to significant biochemical parameters in Supplementary Table 1 (p < 0.2) was used to test independent associations between biochemical parameters and presence of anemia (OR, Odds ratio; 95%CI, 95% confidence interval). Only parameters which remained with p < 0.05 in the adjusted model are shown. Data represent OR per 1Log_10_ increase in values of the parameters.

The overall differences in biomarker values at pre-ATT between anemic and non-anemic TB patients are depicted in Table S1. The study subgroups exhibited statistically significant differences for the evaluations of levels of albumin, and ESR (Table S1). Interestingly, average ferritin levels in both groups were higher than 100 ng/mL, suggesting that TB-associated anemia in the study population is caused by chronic inflammation. We therefore performed an exploratory analysis using hierarchical clustering of z-score normalized data on several biochemical parameters to test if simultaneous assessment of the values of these markers could distinguish anemic from non-anemic individuals (Fig. 2a). We found that pulmonary TB patients with anemia exhibited in general a distinct profile of the biochemical values (Fig. 2a). Although AFB smear grade could not distinguish anemic TB patients from those without anemia (chi-square p = 0.063, Table 2), in the subgroup of anemic TB patients, the hemoglobin levels gradually decreased (Fig. 2b) following increases in AFB smear grade. A step-wise binary multivariate logistic regression analysis demonstrated that increases of 1Log_10_ in albumin values were inversely associated with anemia (adjusted odds ratio [aOR]: 0.22, 95% confidence interval [CI]: 0.07–0.70, p = 0.01) whereas increases in ESR were directly associated with increased odds for anemia (aOR: 1.02, 95% CI: 1.0–1.05, p = 0.04) in the study population (Fig. 2c).

**Figure 3:**
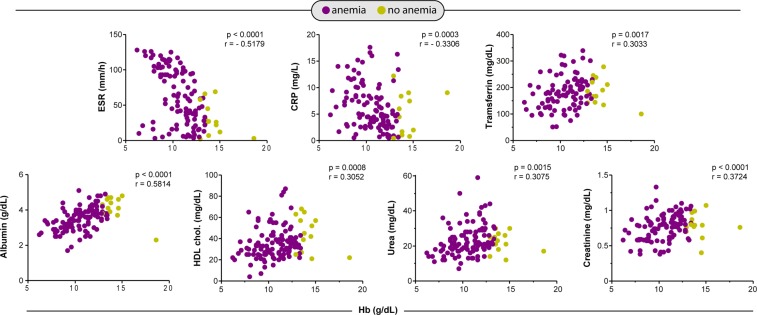
Spearman correlations analysis of biochemical parameters in blood of TB prior to antitubercular treatment initiation. Spearman correlation plots between biochemical parameters and hemoglobin levels are shown. CRP, C-reactive protein; ESR, erythrocyte sedimentation rate; HDL, high-density lipoprotein.

More important than only assessing individual levels of biochemical markers is to examine the relationships between these parameters, which ultimately contribute to a pro-inflammatory or anti-inflammatory environment^3,16^. We thus evaluated the relationships between multiple correlations of the parameters between anemic and non-anemic TB patients. We observed that gradual increases in Hb values were related with remarkable decreases in values of ESR and CRP (Fig. 3). In addition, levels of transferrin, albumin, HDL cholesterol, urea and creatinine were increased proportionally to elevations in Hb values (Fig. 3). These findings clearly indicate that the degree of anemia was directly associated with substantial changes in concentrations of several biochemical parameters that relate to a distinct inflammatory profile in TB patients.

### The inflammatory profile in TB patients with anemia is only partially reverted upon anti-TB therapy implementation

**Figure 4:**
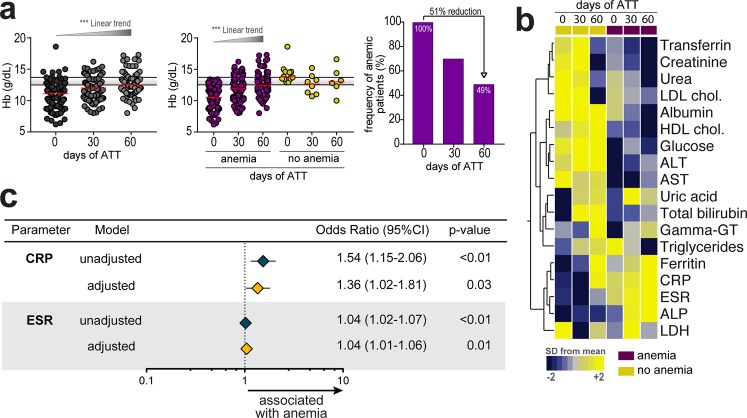
Pulmonary TB patients with anemia persist with systemic inflammatory profile after 60 days of antitubercular treatment initiation. (**a**) Hemoglobin levels at different time points of antitubercular therapy in the entire population as well as in the groups of patients with or without anemia at day 0 of treatment are shown. Data were compared using the Kruskal-Wallis test with Dunn’s multiple comparisons or linear trend post hoc tests. Right panel shows frequency of anemia after initiation of antitubercular therapy in patients with anemia at the study enrollment (day 0 of treatment). ***p < 0.001. Yellow plots represent non-anemic TB patients, purple plots represent anemic TB patients. (**b**) Log10-transformed mean values of the biochemical parameters measured at the different study timepoints were calculated per group of patients according to anemia status at each timepoint. Hierarchical cluster analysis of z-score normalized values illustrates the status of inflammatory profile of TB patients during ATT between those who had anemia or not. (**c**) Logistic linear regression model adjusted to significant biochemical parameters in Supplementary Table 1 (p < 0.2) was used to test independent associations between biochemical parameters measured at pre-ATT and presence of anemia at day 60 of therapy (OR, Odds ratio; 95%CI, 95% confidence interval). Only parameters which remained with p < 0.05 in the adjusted model are shown. Data represent OR per 1Log_10_ increase in values of the parameters.

TB patients were followed up until 2 months after onset of ATT. We had loss to follow-up of 40 patients between day 0 and day 60 (33 were anemic and 7 non-anemic at pre-ATT). When all patients (anemic and nonanemic) who data available were evaluated before and after days 30 and 60 of ATT initiation, we detected gradual increases in Hb values (Fig. 4a). This effect of ATT on Hb levels detected was probably due to changes in patients who were anemic at pre-ATT (linear trend post hoc analysis: p < 0.0001), as Hb concentrations did not change between the study time points in the group who were not anemic at baseline (Fig. 4a). Interestingly, despite the substantial increase in Hb levels upon ATT initiation, the number of anemic individuals at day 60 of therapy was still approximately 49% of the original at pre-ATT (Fig. 4a). Furthermore, hierarchical cluster analysis of the biochemical parameters demonstrated that patients who were anemic at each study timepoint evaluated exhibited with a distinct profile compared to those without anemia (Fig. 4b). We next asked whether baseline anemia was associated with biochemical differences at day 60 of ATT. Noteworthy, TB patients who were anemic at pre-ATT exhibited increased values of ferritin, ESR, CRP and decreased levels of albumin, AST and ALT at day 60 than those who did not have anemia at the study enrollment (Table S2). This finding demonstrates that anemia status prior to anti-TB treatment is associated with altered biochemical profile detected in up to 60 days of ATT. Additional analyses revealed that 13 patients with anemia (21.5%) at day 0 continued to present positive sputum cultures at day 60 of ATT while the same was observed in 7 non-anemic patients (28%). Frequency of culture positive at day 60 was not statistically different between these study groups (p = 0.592).

Finally, we tested if biochemical parameters measured at pre-ATT were able to predict presence of anemia at day 60 of therapy using a model of step-wise logistic regression analysis. We found that increases of 1Log_10_ in values of both CRP (aOR: 1.36, 95% CI: 1.02–1.81, p = 0.03) and ESR (aOR: 1.04, 95% CI: 1.01–1.06, p = 0.01) at pre-ATT were independently associated with presence of anemia at day 60 of therapy (Fig. 4c). These findings reinforce the hypothesis that TB-associated anemia is strongly linked to inflammation in patients undergoing antitubercular treatment.

## Discussion

Anemia is a common condition associated with TB. Several investigations have reported a prevalence of anemia at TB diagnosis ranging between 32% and 86%^12^. Despite its epidemiologic importance, careful investigation of causes of anemia in TB patients is not systematically performed. In the present study, we described the biochemical profile in blood of patients with TB and targeted the identification of profiles associated with mycobacterial loads in sputum as well as response to antitubercular treatment. The results reported here reinforce the idea that anemia is highly prevalent in active TB cases and that most of TB-associated anemia is due to chronic inflammation rather than iron deficiency. Indeed, aside from exhibiting lower levels of hemoglobin, TB patients displayed increased levels of CRP, ESR and uric acid, all of which serve as readouts of systemic inflammation^17^. Nevertheless, in the context of significant inflammation, elevated ferritin levels do not necessarily indicate iron-repletion, as this molecule is also an acute phase reactant and its expression could be driven by inflammation itself ^8–10^. Thus, it is possible that anemia in this context may be due to both chronic disease and iron deficiency. Whether anemia itself promotes changes in several biochemical parameters described here or TB disease progression drives this scenario deserves further investigation in experimental models.

*M*. *tuberculosis* infection burden is known to be linked to inflammatory status^18^. Hemoglobin levels did not change substantially according to increases in AFB smear grade when all patients were considered (Fig. 2b). In addition, AFB smear grade was not different between patients with or without anemia (Table 2). At first glance, these findings could suggest that disease mycobacterial burden does not influence the degree of anemia. However, when only anemic patients were analyzed, we found that hemoglobin levels display a significant linear trend to decrease following gradual increases in AFB smear grades (Fig. 2b). Thus, it is possible that *M*. *tuberculosis* infection burden influences the degree of anemia in more susceptible individuals rather than in every TB patient.

The lowest concentrations of hemoglobin were observed in patients with the highest values of ESR and CRP. Moreover, ESR and CRP concentrations at pre-ATT were independently associated with increased odds for occurrence of anemia at day 60 of therapy, despite an overall improvement of Hb values detected upon anti-TB treatment initiation. Thus, it is possible that increased TB-driven inflammation in newly diagnosed patients is tightly associated with persistent anemia. Previous studies have also reported an inverse correlation between hemoglobin concentrations and ESR in individuals without inflammation or infection^19^, consequently it is not surprising that anemic TB patients reported here would exhibit higher ESR values. A potential explanation for the increased inflammation in TB patients with anemia could be that as the infection progresses, chronic inflammation results in dampened hemoglobin synthesis as reported in anemia of chronic disease^14^. This would mean that anemic TB patients could have longer accumulated duration of *M*. *tuberculosis* multiplication, and thus have been exposed to inflammation for a longer period. The current study recruited patients at the time of disease presentation and for this reason we could not estimate time from onset of active infection to clinical disease. Future studies in endemic areas evaluating recently exposed individuals could be designed to specifically test this hypothesis.

Effective anti-TB treatment is associated with clearance of *M*. *tuberculosis* from sputum. Reduction of mycobacterial load then leads to decreased T-cell activation and its associated inflammatory activity when evaluated in the peripheral blood^20^ but there is also evidence that considerable inflammation may remain or even appear after sputum has turned culture negative^21^. In this setting, monitoring inflammatory status may be useful to predict treatment outcomes. In fact, sustained elevated levels of CRP are observed in TB patients remaining culture positive after 60 days ATT initiation^22^. Whether presence of anemia or systemic inflammation indeed affects the treatment responses or risk of TB relapse is unknown. Herein, in the entire population, median hemoglobin levels significantly changed from day 0 to day 60 of ATT. However, when patients were stratified according to anemia status, we observed that while there was no alteration of hemoglobin levels in nonanemic individuals, substantial elevations were noted in anemic patients. Such effect on anemic patients may be due to successful reduction in TB-driven inflammation during treatment. In addition, anemia has been shown to resolve with anti-TB treatment^23^. Strikingly, at day 60 of ATT, almost 50% of the patients who were anemic at pre-treatment remained with low levels of hemoglobin. This finding indicates that resolution of TB-associated anemia may not occur early after onset of treatment. Using a multivariate logistic regression, we found that individuals with increased levels of CRP and ESR had increased odds for persisting anemia at day 60 of ATT. Of note, we failed to find that frequency of positive sputum cultures at day 60 of ATT was different between anemic and nonanemic patients, but this analysis was underpowered to answer whether the persistent inflammatory profile is at least partially dependent on mycobacterial clearance from sputum, considering the small number of patients with this outcome.

Our study had some limitations similarly to other published reports on TB biomarkers. Because we only had follow-up data until day 60, we were not able to assess associations of anemia with mortality or recurrence/relapse. Hemoglobin values are described to be different in men and women, which may affect the results of this study. The majority of our study population was composed of male individuals, which potentially restricts the inferential power of the results presented here. Additional studies in populations in which female patients are more frequent are necessary to provide definite validation of the findings on evaluation of hemoglobin levels. We did not have samples available to collect information on other iron status markers, such as hepcidin and transferrin receptor (rTRF). Thus, a detailed analysis of inflammation and iron status was not performed. The groups of TB and controls exhibited several differences in overall characteristics, including age, sex and comorbid conditions, which may be related to the fact that the control group was composed by members of the local Fire Department, who may have distinct health status. Nevertheless, such comparisons do not compromise the associations observed within the TB group with or without anemia. Although frequencies of illicit drug use, alcoholism and smoking were not statistically different between TB patients with or without anemia, such conditions may have had an effect on chronic inflammation and hence, potentially, on anemia and additional, larger studies are warranted to test this possibility. Regardless, the findings presented here advance the knowledge in the field by demonstrating a direct association between anemia and persistence of high levels of biomarkers of systemic inflammation upon initiation of antitubercular treatment. In summary, our analyses revealed that TB patients with anemia exhibit a distinct inflammatory profile, which is only partially reverted at day 60 of antitubercular therapy.

## Methods

### Ethics Statement

All clinical investigations were conducted according to the principles expressed in the Declaration of Helsinki. Written informed consent was obtained from each participant at study enrollment. All materials given to the research team were de-identified. The study was approved by the Ethics Committee of the University Hospital of Federal University of Rio de Janeiro (protocol number: 151/05, Ethics committee approval number: 004/05 at 04/28/2005).

### Study Design

The present study is a retrospective assessment of a cohort study that recruited 238 subjects between March 2007 and August 2009, of which 118 individuals with microbiological confirmed pulmonary TB were admitted to a TB referral hospital in the state of Rio de Janeiro, Brazil (Instituto Estadual Ary Pareiras). The cohort included subjects from both sexes, infected or not by HIV (positive serology), with positive smear microscopy or culture for *M*. *tuberculosis* complex subsequently confirmed by biochemical test. The number of TB patients recruited represented 36.2% of all patients hospitalized with a confirmed diagnosis of PTB during the study period. In addition, 120 healthy individuals were recruited as controls (from which, 118 had all epidemiological data available). These controls were healthy volunteers from the Fire Department of the city of Rio de Janeiro and were recruited during a campaign to fight TB. The use of healthy controls was restricted to find the distribution range of normal values of laboratory parameters in Rio de Janeiro. Exclusion criteria for the present analyses were: age under 18 and over 60 years; use of antitubercular drugs at least 1 year before admission; insulin-dependent diabetes mellitus; renal failure and hemodialysis or peritoneal dialysis; blood transfusion under 6 months prior to study enrollment; women in pregnancy or lactation period; and patients whose clinical samples were not subjected to bacteriological or laboratory test. Anemia was defined following WHO criteria as hemoglobin level below 12.5 g/dL for women and 13.5 g/dL for men. Alcohol abuse was investigated using the Cut down, Annoyed, Guilty, and Eye-opener (CAGE) questionnaire^24^. The primary outcome in our study was the proportion of cultures converted to negative at day 60 of treatment, which we associated with anemia, bacteriological status, inflammatory profile and biochemical parameters aspects observed pre-ATT and at day 60 of ATT. Our study population was followed up very closely to assure treatment adherence. During the study period, all pulmonary TB patients included remained hospitalized and received anti-TB drugs under directly observed treatment (DOT). All patients recruited had low-income and low educational status. For these reasons, at the time of the study design, it was decided that all patients would remain hospitalized for 60 days to ensure proper DOT.

### Biochemical measurements

All biochemical measurements were performed in a certified clinical laboratory in Rio de Janeiro. C-reactive protein and transferrin were quantified using nephelometry. Albumin, glucose, triglycerides, total bilirubin, uric acid, high-density lipoprotein (HDL) and low-density lipoprotein (LDL) cholesterol were measured by colorimetric assays. Creatinine, aspartate aminotransferase (AST) and alanine aminotransferase (ALT) were quantified by ultra-violet (UV) kinetics. Urea, ferritin, alkaline phosphatase and gamma-GT were measured using UV urease, enzymatic assay, modified IFCC (International Federation of Clinical Chemistry and Laboratory Medicine) procedure^25^ and Szasz methods^26^, respectively.

### Statistical analysis

The median and interquartile range (IQR) were used as measures of central tendency. Values of biochemical markers were compared between patients stratified according to anemia status using the Mann-Whitney *U* test. Hierarchical cluster analyses with bootstrap (100×) were performed to describe the overall expression profile of biochemical markers in the study population. Fold difference analysis using Holm-Bonferroni’s adjustment for multiple comparisons was employed to identify significant differences for the markers between TB patients and controls, and between anemic and non-anemic TB patients. A model of principal component analysis (PCA) was employed to test which combination of biochemical markers could better distinguish individuals with or without TB diagnosis (pre-treatment). Correlations were assessed using the Spearman rank test. Changes in biomarker values over time of antitubercular therapy were examined using the matched Friedmann test with Dunn’s multiple comparisons or linear trend post hoc test. Two models of logistic regression analysis were performed to test associations between the levels of the biochemical parameters at day 0 or day 60 of antitubercular therapy and presence of anemia at day 60 of treatment. In such multivariable models, data were adjusted to significant biochemical parameters in Supplementary Table 1 (p < 0.2) and tested for independent associations between biochemical parameters and presence of anemia (OR, Odds ratio; 95%CI, 95% confidence interval). Data representing OR per 1Log10 increase in values of the parameters were shown in forest plots. Only parameters which remained with p < 0.05 in the adjusted model are shown in the Figures. All analyses were pre-specified. Two-sided p values of <0.05 were considered statistically significant. Statistical analyses were performed using SPSS 20.0 (IBM statistics), Graphpad Prism 6.0 (GraphPad Software, San Diego, CA) and JMP 12.0 (SAS, Cary, NC, USA).

## Supplementary information

Supplementary Tables

## Data Availability

The datasets generated during and/or analysed during the current study are available from the corresponding author on reasonable request.
