# Supplementary material for: Tuberculosis-associated anemia is linked to a distinct inflammatory profile that persists after initiation of antitubercular therapy
Source: Sci Rep. 2019 Feb 4;9:1381. doi: 10.1038/s41598-018-37860-5 (PMC6361962; doi:10.1038/s41598-018-37860-5)
Supplement: Supplementary file 1 — Supplementary Tables [file 41598_2018_37860_MOESM1_ESM.pdf]

Tuberculosis-associated anemia is linked to a distinct inflammatory profile that persists after initiation of antitubercular therapy

Leonardo Gil-Santana, Luís A. B. Cruz, María B. Arriaga, Pryscila F. C. Miranda, Kiyoshi F. Fukutani, Paulo S. Silveira-Mattos, Elisangela C. Silva, Marina G. Oliveira, Eliene D. D. Mesquita, Anneloek Rauwerdink, Frank Cobelens, Martha M. Oliveira, Afranio Kritski, Bruno B. Andrade

Supplemental Tables

Table S1. Biochemical parameters at pre-ATT

Parameter	Unit	Anemia	No Anemia	p-value
Hemoglobin	g/dL	10.5 (8.9 - 12.3)	13.8 (13.4 - 18.6)	**<0.01**
Transferrin*	mg/dL	176.5 (120 - 230)	186 (134 - 278)	0.65
Ferritin*	ng/mL	334.5 (106 - 610.1)	387 (164.6 - 577)	0.79
ESR	mm/h	59 (18 - 105)	26 (3 - 69)	**<0.01**
CRP	mg/L	5 (2.5 - 10.6)	4.5 (0.7 - 12.2)	0.26
HDL cholesterol	mg/dL	33 (25 - 49)	45 (22 - 68)	**0.05**
LDL cholesterol	mg/dL	82 (63 - 109)	75 (57 - 126)	0.87
Triglycerides	mg/dL	89 (61 - 124)	105 (71 - 177)	0.25
Glucose	mg/dL	92 (82 - 111)	99 (75 - 205)	0.22
Albumin	g/dL	3.5 (2.9 - 4.1)	4.3 (3.7 - 4.8)	**<0.01**
Total Bilirubin**	mg/dL	0.3 (0.2 - 0.7)	0.4 (0.2 - 1.5)	0.27
AST	U/L	26 (18 - 50)	29 (18 - 386)	0.29
ALT	U/L	21 (12 - 43)	28 (12 - 201)	**0.13**
ALP	U/L	103 (67 - 196)	88 (62 - 180)	0.29
Gamma-GT	U/L	104 (49 - 204)	132 (50 - 361)	0.51
LDH	U/L	174 (140 - 223)	178 (131 - 420)	0.87
Acid Uric	mg/dL	9.1 (6.2 - 12.4)	10 (5.1 - 15.9)	0.41
Urea	mg/dL	21 (18 - 31)	23 (14 - 30)	0.84
Creatinine	mg/dL	0.8 (0.6 - 0.9)	0.8 (0.7 - 1.1)	0.82

Data represent median and interquartile ranges of markers measured at day 0 of antitubercular treatment in the subgroup of patients who had anemia or not at the study baseline. Variables were compared using the Mann-Whitney U test. ALP, alkaline phosphatase; ALT, Alanine Transaminase; AST, Aspartate Aminotransferase; Gamma-GT, Gamma-glutamyl Transferase; HDL, High-density Lipoprotein; LDH, Lactate Dehydrogenase; LDL, Low-density; Lipoprotein MCH, Mean Cell Hemoglobin; MCV, Mean Cell Volume; MCHC, Mean Cell Hemoglobin Concentration; RDW, Red Cell Distribution Width; VLDL, Very-low-density Lipoprotein. Variables with asterisks presented different number of patients from which data was available for, *information from 105 patients was available, **information from 117 patients was available

Table S2. Biochemical parameters at day 60 of therapy

Parameter	Unit	Anemia	No Anemia	p-value
Hemoglobin	g/dL	11.7 (10.9 - 12.8)	14.4 (13.4 - 16.6)	**<0.01**
Transferrin*	mg/dL	191 (152 - 238)	221.5 (160 - 243)	0.06
Ferritin*	ng/mL	223.9 (47.6 - 443.8)	81.2 (26 - 226.9)	**0.01**
ESR	mm/h	39 (16 - 93)	17.5 (2 - 62)	**<0.01**
CRP	mg/L	3.4 (1.2 - 9.1)	1.3 (0.1 - 3.6)	**<0.01**
HDL cholesterol	mg/dL	36 (30 - 49)	39 (31 - 65)	0.14
LDL cholesterol	mg/dL	88 (74 - 114)	93 (60 - 125)	0.96
Triglycerides	mg/dL	111 (74 - 157)	92 (61 - 154)	0.07
Glucose	mg/dL	91.5 (86 - 134)	98 (85 - 162)	0.12
Albumin	g/dL	4.1 (3.7 - 4.6)	4.3 (3.9 - 4.6)	**0.04**
Total Bilirubin**	mg/dL	0.3 (0.2 - 0.4)	0.3 (0.2 - 0.5)	0.66
AST	U/L	23.5 (18 - 36)	31 (19 - 47)	**0.01**
ALT	U/L	16 (10 - 31)	24 (15 - 46)	**<0.01**
ALP	U/L	92.5 (66 - 130)	86.5 (57 - 119)	0.15
Gamma-GT	U/L	72.5 (38 - 186)	83 (42 - 186)	0.98
LDH	U/L	170 (137 - 210)	187.5 (151 - 241)	0.07
Acid Uric	mg/dL	7.4 (4.2 - 12)	6 (4 - 11.6)	0.21
Urea	mg/dL	25 (18 - 40)	28 (20 - 35)	0.48
Creatinine	mg/dL	0.8 (0.6 – 1.0)	0.9 (0.6 - 1.1)	0.19

Data represent median and interquartile ranges of markers measured at day 60 of antitubercular treatment in the subgroup of patients who had anemia or not at the study baseline (day 0). Variables were compared using the Mann-Whitney *U* test. ALP, alkaline phosphatase; ALT, Alanine Transaminase; AST, Aspartate Aminotransferase; Gamma-GT, Gamma-glutamyl Transferase; HDL, High-density Lipoprotein; LDH, Lactate Dehydrogenase; LDL, Low-density; Lipoprotein MCH, Mean Cell Hemoglobin; MCV, Mean Cell Volume; MCHC, Mean Cell Hemoglobin Concentration; RDW, Red Cell Distribution Width; VLDL, Very-low-density Lipoprotein. Variables with asterisks presented different number of patients from which data was available for, *information from 105 patients was available, **information from 117 patients was available
